# Ginseng alleviates cyclophosphamide-induced hepatotoxicity *via* reversing disordered homeostasis of glutathione and bile acid

**DOI:** 10.1038/srep17536

**Published:** 2015-12-02

**Authors:** He Zhu, Min-Hui Long, Jie Wu, Meng-Meng Wang, Xiu-Yang Li, Hong Shen, Jin-Di Xu, Li Zhou, Zhi-Jun Fang, Yi Luo, Song-Lin Li

**Affiliations:** 1 Department of Pharmaceutical Analysis, Affiliated Hospital of Integrated Traditional Chinese and Western Medicine, Nanjing University of Chinese Medicine, Nanjing 210046, PR China; 2 Department of Metabolomics, Jiangsu Province Academy of Traditional Chinese Medicine, Nanjing 210028, PR China; 3 College of Biotechnology, Tianjin University of Science and Technology, Tianjin 300222, PR China; 4 Clinical Pharmacology Laboratory, The Second Affiliated Hospital of Soochow University, Suzhou 215004, PR China; 5 Department of Oncology, Affiliated Hospital of Integrated Traditional Chinese and Western Medicine, Nanjing University of Chinese Medicine, Nanjing 210046, PR China

## Abstract

Cyclophosphamide (CP), a chemotherapeutic agent, is restricted due to its side effects, especially hepatotoxicity. Ginseng has often been clinically used with CP in China, but whether and how ginseng reduces the hepatotoxicity is unknown. In this study, the hepatoprotective effects and mechanisms under the combined usage were investigated. It was found that ginseng could ameliorate CP-induced elevations of ALP, ALT, ALS, MDA and hepatic deterioration, enhance antioxidant enzymes’ activities and GSH’s level. Metabolomics study revealed that 33 endogenous metabolites were changed by CP, 19 of which were reversed when ginseng was co-administrated *via* two main pathways, i.e., GSH metabolism and primary bile acids synthesis. Furthermore, ginseng could induce expression of GCLC, GCLM, GS and GST, which associate with the disposition of GSH, and expression of FXR, CYP7A1, NTCP and MRP 3, which play important roles in the synthesis and transport of bile acids. In addition, NRF 2, one of regulatory elements on the expression of GCLC, GCLM, GS, GST, NTCP and MRP3, was up-regulated when ginseng was co-administrated. In conclusion, ginseng could alleviate CP-induced hepatotoxicity *via* modulating the disordered homeostasis of GSH and bile acid, which might be mediated by inducing the expression of NRF 2 in liver.

Cyclophosphamide (CP), an oxazaphosphorine derivative of the classical alkylating agent nitrogen mustard, has been widely used to treat various forms of cancers, including lymphoma, breast cancer and leukemia^1^,^2^, for more than 50 years. However, clinical application of CP is often restricted due to its serious side effects^3^, especially hepatotoxicity^4^,^5^. Metabolic conversion of CP could lead to the formation of several kinds of cytotoxic metabolites^6^, which might induce oxidative stress and cause the hepatotoxicity. For example, acrolein (AC)^7^, a highly reactive metabolite of CP with short biological half-life, could readily react with glutathione (GSH), one of thiol containing proteins, which serves several vital functions, including detoxifying electrophiles and relieving oxidative stress, with the presence of glutathione S-transferase (GST)^8^. However, when GSH was exhausted, AC, the highly reactive α, β-unsaturated aldehyde, could react with cellular nucleophiles^7^,^9^, such as the thiol groups of cysteine residues in proteins and nitrogen atoms in lysine and histidine groups, leading to the loss of protein function, which could induce the oxidative stress and finally give rise to the disastrous effects on hepatocyte.

At present, several therapeutic strategies, including combination of several chemotherapeutic drugs in low doses^10^ and application of alternative analogues of CP^11^, have been proposed as possible approaches for the prevention of CP-induced side effects. However, the clinical outcomes are not satisfactory, since a significant percentage of patients received combinative chemotherapy or CP analogs still suffer from liver dysfunction^12^,^13^, and discontinuation of CP therapy remains the best option in cases of progressive liver damage. Therefore, finding an effective way to prevent CP-induced hepapotoxicity is a critical issue during the clinical application of CP.

Ginseng, one of the most famous traditional Chinese medicines, has been commonly used as a tonic and panacea food during radiotherapy and chemotherapy in many countries and regions, especially in East Asia. Many groups had reported that ginseng and ginsenosides, the active components of ginseng^14^, could elevate the GSH level *via* promoting the expression of glutamate cysteine ligase (GCL), the rate-limiting step of GSH. Current researches indicated that ginseng and ginsenosides could reduce the side effects, including genotoxicity, cell apoptosis^15^,^16^, reproductive toxicity^17^ and oxidative stress^18^, and enhance the anti-cancer effect of CP^15^,^19^,^20^,^21^. Recently, one preparation named Compound Cyclophosphamide, the main ingredients are CP and total ginsenosides from ginseng, has been clinically used in China^16^. However, up to date, whether ginseng could alleviate the CP-induced hepaptotoxicity and its relevant mechanisms were still unknown. Given this situation, it is an important issue to evaluate the effect of ginseng on CP-induced hepaptotoxicity for rationalization of clinical application of ginseng combined with CP.

Metabolomics is the comprehensive assessment and simultaneous profiling of endogenous metabolic changes in living systems^22^, which could offer global variations of low molecular weight metabolites in biological samples and generate valuable information about the physiological changes^23^. With the development of analysis and data process, the metabolomics has become one of the most powerful tools to find the most susceptible metabolic pathways, which could supply important information during the finding of potential targets^24^; on the other hands, traditional molecular biotechnology could illuminate the intricate variations focused on one or more targets. Metabolomics and molecular biotechnological methods could complement each other, and it might show convenience during the main mechanism study if the two methods were combined.

In the present study, the hepatoprotective effects of ginseng on CP-induced hepatotoxicity and the potential mechanisms involved were investigated with metabolomics approach together with molecular biotechnology, so as to rationalize the combinative application of ginseng and CP in the cancer therapy.

## Results

### Effects of ginseng on the behavior and body weight in CP treated rats

**Figure 1:**
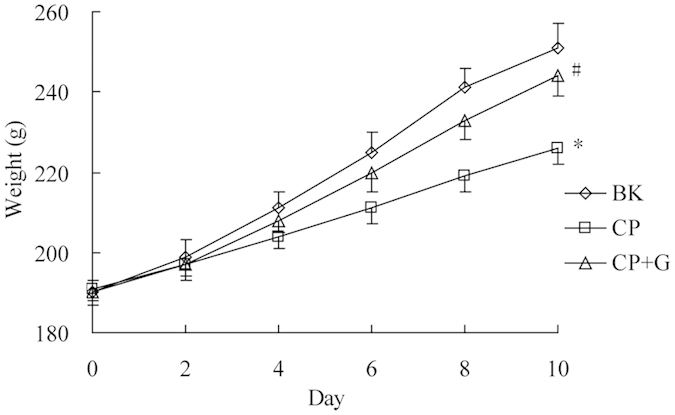
Influence of ginseng on the body weight of CP treated rats (n = 6). BK for blank group, CP for CP treated group and CP+G for CP and ginseng co-treated group. Compared with BK, *p < 0.05; compared with CP, ^#^p < 0.05.

The rats in CP treated group appeared exhausted, sluggish, asthmatic and somnolent symptom, while the symptom was relieved when ginseng was coadministrated. The effects of ginseng on the body weight of CP-induced rats are shown in Fig. 1. The rats which were treated by CP showed a significant reduction in the body weight (p < 0.05) compared with blank group on the 10th day. However, rats in CP and ginseng co-treated group showed significant enhancement in the body weight when compared to that in the CP treated group.

### Effects of ginseng on aminotransferase activities in CP treated rats

The aminotransferase activities in serum are shown in Table 1. The markers of hepatic damage components alkaline phosphatase (ALP), aspartate transaminase (AST) and alanine transaminase (ALT) were elevated 2.45, 2.74 and 2.08 folds in CP treated group compared with blank group. However, when ginseng was used, CP-induced elevations of ALP, AST and ALT were markedly reduced.

### Effect of ginseng on histopathology of liver in CP treated rats

**Figure 2:**
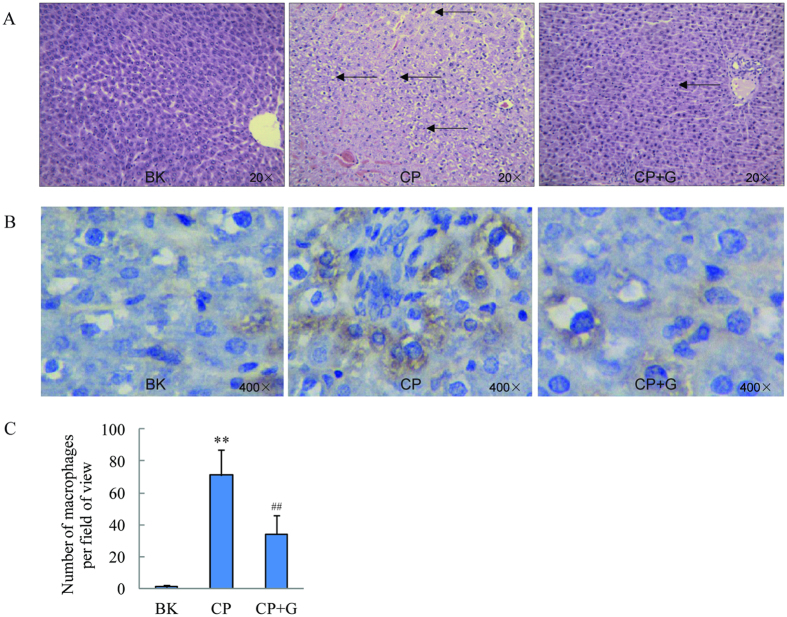
Micrograph of a section of rat liver. (**A**) H&E stained histological features (20×) of rats’ liver, (**B**) F4/80 stained histological features (400×) of rats’ liver and (**C**) number of macrophage infiltrating into liver (n = 5). BK for blank group, CP for CP treated group and CP+G for CP and ginseng co-treated group.

Hematoxylin and eosin (H&E) staining examination (Fig. 2A) was performed on the liver for any abnormalities on the structure of the hepatocytes. The livers samples obtained from blank group showed normal architecture and normal hepatic cells with well preserved cytoplasm and well defined cell lining and round nuclei. The sections of CP treated liver demonstrated extensive area of inflammation and ballooning fatty degeneration or steatosis. However, the pathological changes in CP and ginseng co-treated group were not as serious as CP treated group.

### Effect of ginseng on macrophage infiltration of liver in CP treated rats

To investigate the effects of ginseng on macrophage infiltration (associated with acute inflammation) induced by CP, immunostaining for F4/80^25^,^26^, a macrophage marker in rat, was performed (Fig. 2B,C). Cells positive for F4/80 were present in the liver of CP treated group, whereas, the number of such cells was reduced significantly when ginseng was co-treated.

### Effects of ginseng on oxidative damage in CP treated rats

The malondialdehyde (MDA) and GSH concentrations and the antioxidant enzymes activities in liver tissues are presented in Table 1. The concentration of MDA, an end product of lipid peroxidation (LPO), was significantly increased by 1.97 fold, and GSH content was decreased by 42.6% in CP treated group compared with blank group. In addition, the activities of antioxidant enzymes, which were superoxide dismutase (SOD), catalase (CAT) and glutathione peroxidase (GPO), and drug detoxifying enzyme (GST) were significant decreased by 42.6%, 19.4%, 28.3%, 26.4% and 51.2% with the activity of glutathione reductase (GR) increased by 13.8% after treatment of CP when compared with blank group. In contrast, the concentration of MDA in the ginseng and CP co-treated group was significantly decreased compared with that in the CP group, whereas the content of GSH and the activities of SOD, GPO GST were significantly increased. Besides, the activities of CAT and GR were also returned in ginseng and CP co-treated group compared with CP treated group, although without significant difference.

### Effects of ginseng on inflammatory mediators in CP treated rats

The levels of inflammatory mediators in serum are listed in Table 1. Interleukine-6 (IL-6) and Interleukine-8 (IL-8) were elevated 3.3 and 3.2 folds while Interleukine-10 (IL-10) was reduced to 0.34 time in CP treated group compared with blank group. However, when ginseng was co-administrated, CP-induced variations of inflammatory mediators were markedly reversed, although only IL-6 and IL-10 with statistical differences.

### Metabolomics study of ginseng against CP-induced hepatotoxicities

#### Data acquisition and identification of the endogenous biomarkers

**Figure 3:**
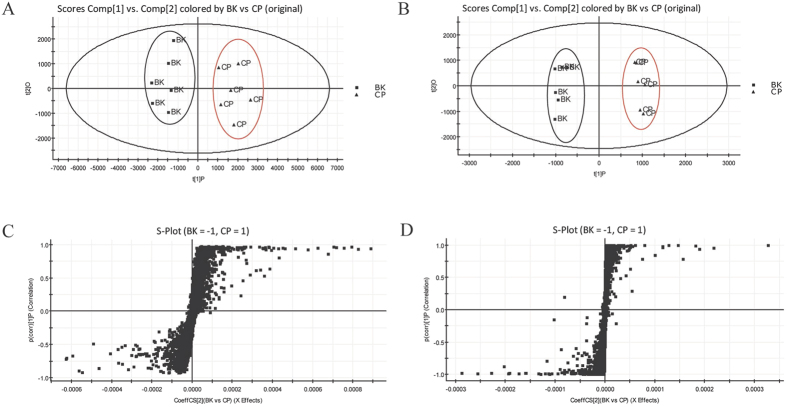
The serum PCA score plot and S-plot of OPLS-DA between blank and CP treated groups. (**A**) PCA score plot in positive ion mode, (**B**) PCA score plot in positive ion mode, (**C**) S-plot of OPLS-DA in positive mode and (**D**) S-plot of OPLS-DA in positive mode. BK for blank group and CP for CP treated group.

A clear separation in the principal component analysis (PCA) between blank group and CP treated group was obtained (Fig. 3A,B), which indicated that they had different endogenous biomarkers. From S-plot of orthogonal partial least squares-discriminant analysis (OPLS-DA, Fig. 3C,D), endogenous biomarkers which had significant differences between blank and CP groups could be found. By setting the variable importance in the projection (VIP) value at >1, 172 potential biomarkers were detected in serum, including 92 in ESI positive-ion mode and 80 in ESI negative-ion mode. The potential biomarkers of interest were extracted from loading-plots and S-plots constructed following analysis with partial least-squares-discriminant analysis (PLS-DA) and OPLS-DA from MarkerLynx, respectively. The *m/z* values and the exact mass of the metabolites were obtained. Several online databases, such as HMDB (http://www.hmdb.ca/) and KEGG (http://www.kegg.jp/), were used for initial determination of the metabolites. The mass tolerance between the measured *m/z* value and the exact mass of the components of interest was set to within 20 mDa. The MS/MS spectra of significantly different metabolites were obtained using a targeted MS/MS mode. And the metabolites were identified by comparing the MS/MS spectra of the analytes with that in the databases or literatures.

According to the protocol detailed above, 33 endogenous biomarkers (Table 2) in serum samples were tentatively identified according to their molecular ion information and corresponding fragments of product ion. Here, the endogenous metabolite M1 with T_R_-*m/z* of 9.25–524.3712 in positive ion mode was detailed as an example to illustrate the identification process. Firstly, the accurate mass of the potential marker was determined: its corresponding peak was made out according to its retention time in total ion chromatogram from ESI positive-ion scan mode, then an accurate mass of the marker ([M + H]^+^ at *m/z* 524.3712) was found from the mass spectrum. Secondly, particular MS/MS information about fragmentation pattern of the marker was acquired from QTOF system. In the positive ion spectrum, the main fragment ions of the marker were observed at *m/z* 506.3451, 341.3052, 184.0737 and 104.1073, which could be the loss of H_2_O, C_5_H_14_NO_4_P, C_21_H_40_O_3_ and C_21_H_41_O_6_P in [M + H]^+^ respectively. Using a mass tolerance of 20 mDa, C_26_H_54_NO_7_P was located as the candidate because its high mass accuracy among the possible compounds. Finally, the metabolite M1 was identified as lysophosphatidylcholine (18:0) [lysoPC (18:0)] according to the HMDB and KEGG database.

#### Influence of ginseng on the endogenous biomarkers in CP treated rats

**Figure 4:**
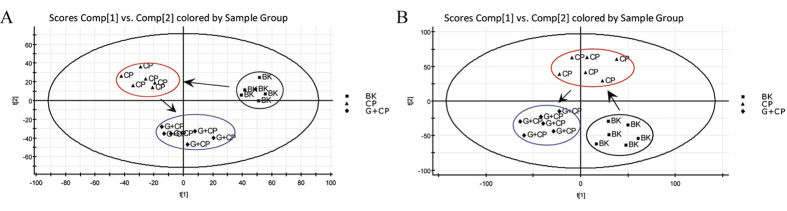
The serum PLS-DA score plot among blank, CP treated and CP and ginseng co-treated groups. (**A**) positive mode and (**B**) negative mode. BK for blank group, CP for CP treated group and CP+G for CP and ginseng co-treated group.

In order to examine whether ginseng could influence the CP-induced endogenous biomarkers in rats, PLS-DA was conducted. On days 10 (Fig. 4A,B), a clear separation among blank, CP treated and CP and ginseng co-treated groups from score plot of the PLS-DA was easily seen. Additionally, in order to more clearly characterize the protective effect of ginseng, relative intensity of the endogenous biomarkers in Table 2 were analyzed for semi-quantitation, and it was found that the contents of main key biomarkers in CP and ginseng co-treated group was closer to blank group in comparison with CP treated group. Among the most affected 33 endogenous biomarkers by CP, 19 endogenous biomarkers were reversed significantly according to the parametric t-test and 12 endogenous biomarkers were also returned without statistical difference after the co-administration of ginseng.

#### Influence of ginseng on the metabolic pathways in CP treated rats

**Figure 5:**
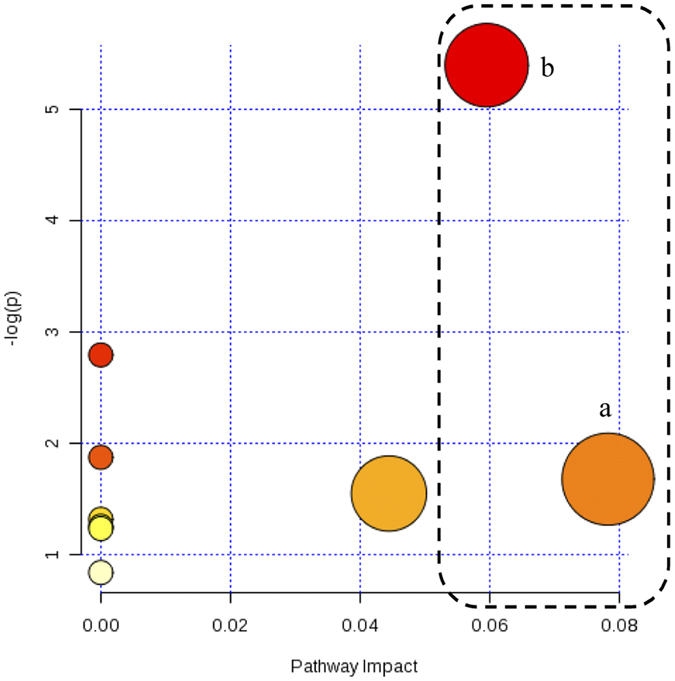
Summary of pathway analysis of serum samples of CP and ginseng co-treated rats. a: Primary bile acid biosynthesis and b: Glutathione metabolism.

In order to explore the possible metabolic pathways that were affected by ginseng on the hepatotoxicity induced by CP, 19 significantly reversed endogenous biomarkers by ginseng were imported into MetaboAnalyst. Nine metabolic pathways which were primary bile acid biosynthesis, taurine and hypotaurine metabolism, sphingolipid metabolism, glycerophospholipid metabolism, GSH metabolism, fatty acid metabolism, biosynthesis of unsaturated fatty acids, fatty acid biosynthesis and steroid hormone biosynthesis were constructed (Fig. 5). All of the nine metabolic pathways are related to the function of liver, but only primary bile acid biosynthesis with the impact-value 0.05952 and GSH metabolism with the impact-value 0.07824 were filtered out as the most important metabolic pathways, as the pathway with the impact-value threshold above 0.05 was regarded as potential target pathway. Therefore, it can be referred that ginseng alleviates the CP-induced hepatotoxicity *via* modulating the disordered homeostasis of primary bile acid and GSH *in vivo*.

### Effects of ginseng on the expression associated with GSH homeostasis in CP treated rats

**Figure 6:**
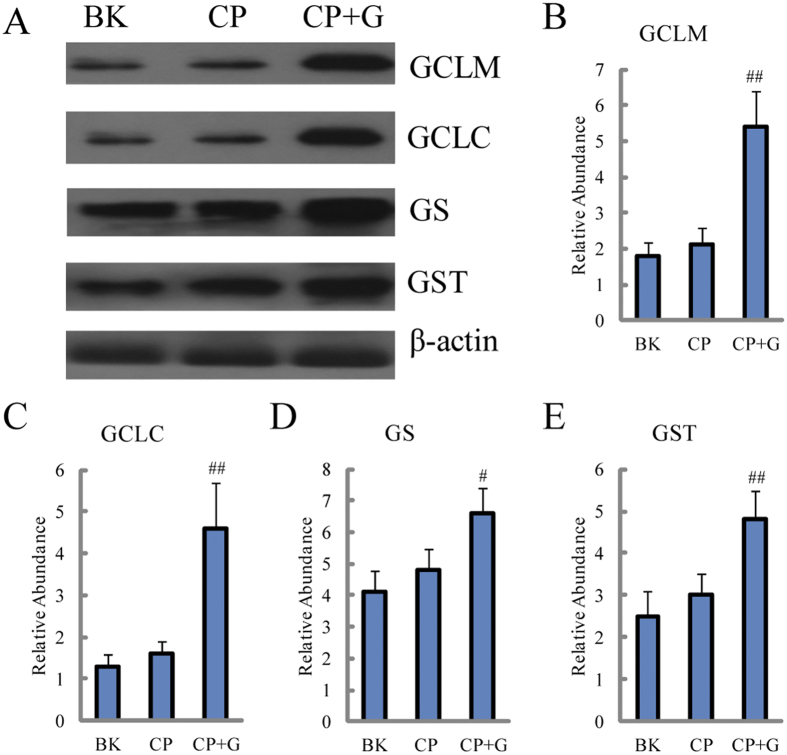
Protein expressions related to the disposition of GSH. (**A**) western blot analysis of proteins associated with the synthesis and excretion of GSH in rats’ liver treated by CP with the presence and absence of ginseng. Detection of β-actin expression was used as a loading control. The bar graphs show quantitative relative level of (**B**) GCLM, (**C**) GCLC, (**D**) GS and (**E**) GST protein expressions for blank, CP treated and CP and ginseng co-treated rats. Values are presented as means ± SD (n = 6). BK for blank group, CP for CP treated group and CP+G for CP and ginseng co-treated group. Compared with CP, ^#^p < 0.05 and ^##^p < 0.01.

From metabolomics results, GSH level is an important contributor to the protective effect of ginseng on the hepatotoxicity induced by CP. The GSH level could affect the homeostasis of oxidation and reduction *in vivo*. Therefore, the relative levels of key protein involved in GSH biosynthesis, which were glutamate cysteine ligase modifier subunit (GCLM), glutamate cysteine ligase catalytic subunit (GCLC) and GSH synthase (GS), and transfer enzyme (GST) were further studied. As shown in Fig. 6, the expressive levels of the target proteins were slightly increased in CP group compared with blank group, however, dramatically elevated with significant statistical differences when ginseng was co-administrated.

### Effects of ginseng on the expression associated with bile acids homeostasis in CP treated rats

**Figure 7:**
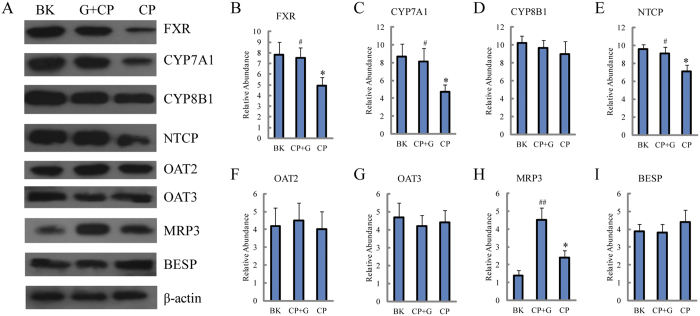
Protein expressions related to the disposition of bile acid. (**A**) western blot analysis of proteins associated with the synthesis, transport and excretion of bile acid in rats’ liver treated by CP with the presence and absence of ginseng. Detection of β-actin expression was used as a loading control. The bar graphs show quantitative relative level of (**B**) FXR, (**C**) CYP7A1, (**D**) CYP8B1, (**E**) HTCP, (**F**) OAT2, (**G**) OAT3, (**H**) MRP3 and (**I**) BESP protein expressions for blank, CP treated and CP and ginseng co-treated rats. Values are presented as means ± SD (n = 6). BK for blank group, CP for CP treated group and CP+G for CP and ginseng co-treated group. Compared with BK, *p < 0.05; compared with CP, ^#^p < 0.05 and ^##^p < 0.01.

On the basis of the metabolomics results, the alleviation of ginseng on the CP-induced hepatotoxicity was highly related to the primary bile acid network. Therefore, the relative level of key proteins involved in bile acid biosynthesis (farnesoid X receptor (FXR), cholesterol 7-alpha-hydroxylase (CYP7A1) and sterol 12-α-hydroxylase (CYP8B1)), uptake (Na^+^-taurocholate-cotransporting protein (NTCP), organic anion transporters 2 (OAT 2) and organic anion transporters 3 (OAT 3)) and excretion (multi-drug resistance-associated protein 3 (MRP 3) and bile salt export pump (BSEP)) was further confirmed. As shown in Fig. 7, the levels of FXR, CYP7A1 and NTCP were significantly decreased, whereas the level of MRP 3 was significantly increased in CP treated group compared with blank group. However, when ginseng was co-treated, the expression levels of FXR, CYP7A1, NTCP and MRP3 were significantly increased compared with those in the CP treated group.

### Effects of ginseng on the expression of nuclear factor erythroid 2-related factor 1 (NRF 1) and nuclear factor erythroid 2-related factor 2 (NRF 2) in CP treated rats

**Figure 8:**
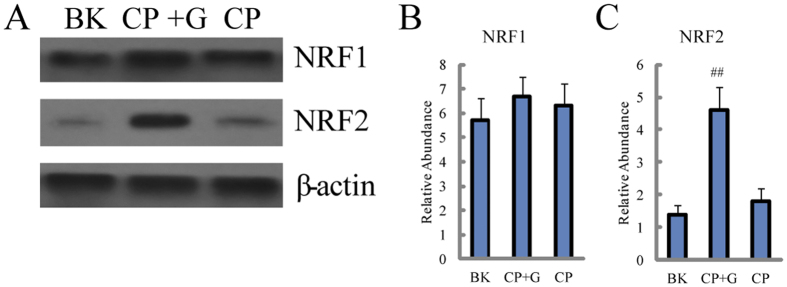
Protein expressions of NRF 1 and NRF 2 (A) western blot analysis of protein expressions of NRF 1 and NRF 2 in rats’ liver treated by CP with the presence and absence of ginseng. Detection of β-actin expression was used as a loading control. The bar graphs show quantitative relative level of (**B**) NRF 1 and (**C**) NRF 2. Values are presented as means ± SD (n = 6). BK for blank group, CP for CP treated group and CP+G for CP and ginseng co-treated group. Compared with CP, ^##^p < 0.01.

NRF 1 and NRF 2, the most important nuclear receptors, could regulate the expression of several enzymes and transporters which were related to the expression of GSH and bile acids, including GCLC, GCLM, GS, GST, NTCP and MRP3. Thus, we further investigated the expression of NRF 1 and NRF 2 when ginseng was co-administrated with CP. As shown in Fig. 8, the expression of NRF 1 and NRF 2 were slightly elevated without significant difference in CP group compared with blank group, however, when ginseng was co-treated, the expressive level of NRF 2 was significantly increased compared with those in the CP treated group.

### Role of NRF 2 in ginseng mediated hepatoprotective effect in CP treated rats

**Figure 9:**
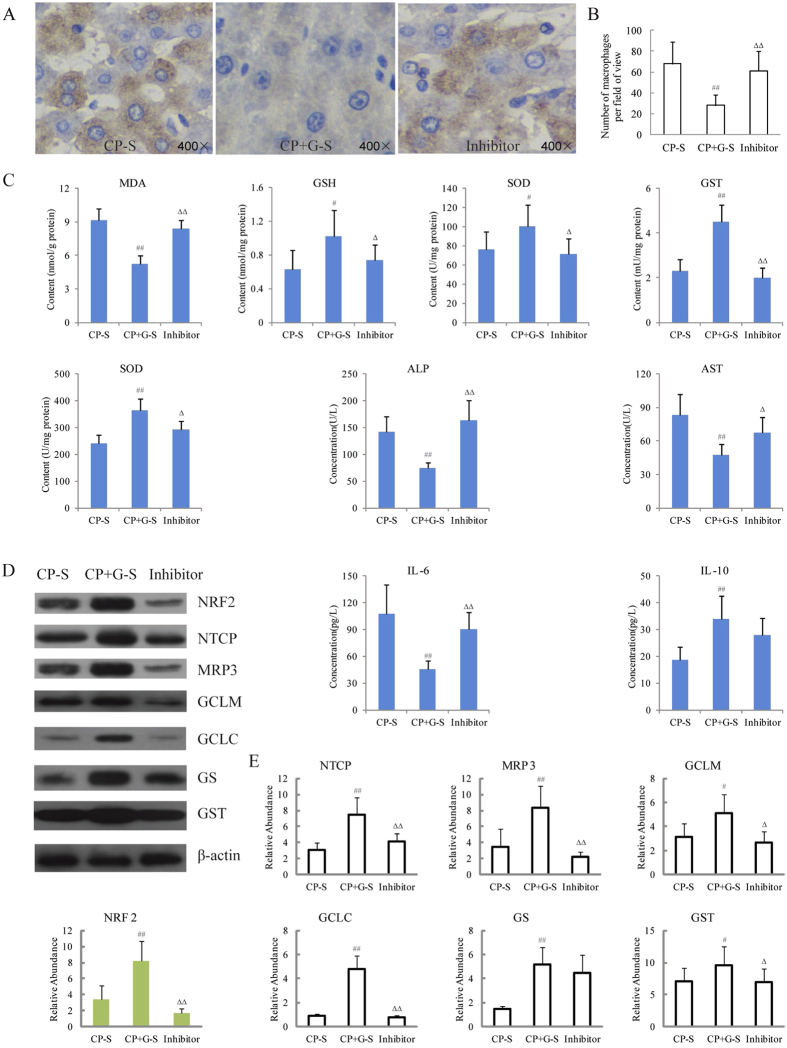
Effect of NRF 2 inhibitor on ginseng mediated hepatoprotective effect induced by CP. (**A**) F4/80 stained histological features (400×) of rats’ liver with the inhibitor, (**B**) number of macrophage infiltrating into liver with the inhibitor (n = 5), (**C**) biochemical parameters with the inhibitor, (**D**) and (**E**) western blot analysis of protein expressions and relative levels in rats’ liver mediated by ginseng with the inhibitor. Values are presented as means ± SD (n = 6). CP-S for CP satellite group, CP+G-S for CP and ginseng co-treated satellite group and inhibitor for inhibitor group. Compared with CP-S, ^#^p < 0.05, ^##^p < 0.01; compared with CP+G-S, ^△^p < 0.05 and ^△△^p < 0.01.

To investigate the role of NRF 2 on the hepatoprotective effect of ginseng in CP treated rats, Brusatol, a specific NRF 2 inhibitor^27^,^28^, was selected to research the infiltration of macrophage, the levels of biochemical indicators and the expressions of related proteins which were altered significantly between CP treated rats and CP and ginseng co-treated rats. As shown in Fig. 9, when NRF 2 was inhibited, ginseng caused the abirritation of macrophage infiltration and the reverse phenomena of biochemical indicators levels and proteins expression were nearly abrogated with significant difference or slightly attenuated. This observation provided evidence that the NRF 2 might be involved in the hepatoprotective effect of ginseng in CP treated rats.

## Discussion

Oxidative stress, caused by the active metabolites of CP, exhibited a catastrophic effect on hepatocellular membranes and destructive capability of target tissues. Therefore, finding a strategy to reduce the oxidative stress might grasp a key, at least in part, to alleviate the CP-induced hepatotoxicity. Recently, it was reported that ginseng, shows anti-oxidative effects, could inhibit liver dysfunction during the exposure of some hepatotoxic materials^29^, including acetaminophen, whose metabolites could induce oxidative stress^30^. Thus, we raised a hypothesis to which ginseng might have the ability to alleviate the CP-induced hepatotoxicity *via* alleviating oxidative stress. Besides, combination of CP and ginseng or ginsenosides, which had been clinically applied for many years not only in Asian countries but also in Western countries, showed synergistic anticancer effects^31^,^32^. Based on that fact, it is necessary to illuminate the influence of ginseng on CP-induced hepaptotoxicity and further investigate the relevant mechanisms, which will replenish the theoretical foundation during the clinical combination of CP and ginseng.

In the present study, CP-induced apparent heptatotoxic (but not lethal) phenomena, which were consistent with previous reports^33^,^34^, were appeared, including elevated liver damage indexes (ALT, AST and ALP) and disturbed inflammatory mediators^35^,^36^ (IL-6, IL-8 and IL-10) in serum, infiltrated inflammatory cells (macrophages) and heavy hepatic steatosis in liver, as well as reduced tendency of body weight. Whereas, when ginseng was used, CP-induced reductive tendency of body weight was alleviated, elevated ALT, AST and ALP levels were degraded, imbalanced IL-6, IL-8 and IL-10 were reversed, infiltrated macrophages were nearly disappeared and degenerated hepatocytes were attenuated, respectively. All of above evidences indicated the protective effect of ginseng on CP-induced hepatotoxicity.

Based on previous studies, oxidative stress is one of the principal causes of CP-induced hepatotoxicity and is mediated by the production of CP’s metabolites. As expected, in our study, CP treatment resulted in increasing MDA concentration with decreasing GSH level and SOD, CAT, GST, GPO and GR activities. Whereas, ginseng prevented the CP-induced depletion of GSH level, suppression of SOD, GST and GPO activities, and increment of MDA concentration in liver. These results indicate clearly that co-treatment with ginseng protected effectively against oxidative stress induced by CP.

Liver is a multifunctional organ which is involved in various enzymatic metabolic activities. Damage to this important organ by exogenous hepatotoxic agent may lead to the disturbance of endogenous metabolites^37^,^38^,^39^,^40^,^41^, such as fatty acids, amino acids and bile acids, which could be surveyed by metabolomics. Accordingly, 33 endogenous metabolites were found quantitatively changed by CP treatment, and 19 biomarkers, which were associated with nine metabolic pathways and related with the function of liver, were returned when ginseng was combined with CP. Among the constructed pathways, GSH metabolism and primary bile acid biosynthesis were filtered out to be the most important metabolic pathways. The results suggested that modulating the disordered homeostasis of GSH and primary bile acid were the key factors in the protective effects of ginseng on CP-induced hepatotoxicity. Therefore, in the current study, the protein expression associated with the biosynthesis and disposition of GSH and bile acids were further investigated.

Numerous data confirmed that CP-induced hepatotoxicity was associated with, at least in part, the level of GSH, which played an important role in eliminating active metabolites and defending the oxidative stress *in vivo*^42^. GSH was synthesized by amino acids *via* two ATP-requiring enzymatic steps in the cytosol of cells. The first step is considered to be the rate-limiting step and catalyzed by GCL, which is composed by GCLC, the catalytic subunit, and GCLM, the modifier subunit, whereas, the second step is catalyzed by GS^42^,^43^. However, in our study, it was interesting to find that the expressions of GCLC, GCLM and GS were slightly elevated, although without statistical difference, and the GSH level was nearly exhausted in CP treated group. The reasons might be that GCLC, GCLM and GS were induced by several electrophilic electron-deficient metabolites, which could react with GSH quickly with the presence of GST. For example, it was reported that AC, one of the active metabolites of CP, is a kind of α, β-unsaturated aldehydes, could not only promote the expression of GCLC, GCLM and GS, but also eliminate the GSH in a few minutes. In addition, the activity of GST was significant inhibited, but the expression was not reduced synchronously in CP treated group, which was also observed in previous reports^44^,^45^. We assumed that when GSH was exhausted, electrophilic electron-deficient metabolites might covalently bond with the thio in the GST in a short time and finally inhibit the activity of GST. Whereas, when ginseng was co-administrated, the expressions of GCLC, GCLM, GS and GST were up-regulated dramatically with the elevation of GSH and activation of GST. Therefore, we assume that the hepatoprotective mechanism of ginseng against CP-induced oxidative stress is partially mediated by elevating the GSH level and promoting the generation of CP metabolite-GSH conjugations.

Bile acid was observed for the first time to be another key factor in CP-induced hepatotoxicity. Bile acids are the main endogenous ingredients in bile, which are synthesized and secreted by hepatcyctes into the bile canaliculus. Oxidative stress could disturb the homeostasis of bile acids, whereas, disordered homeostasis of bile acids could aggravate the oxidative stress of liver^37^,^38^,^40^,^46^. Besides, unbalanced composition of bile acids can directly induce the hepatotoxicity *via* several pathways, including proinflammatory and cytotoxicity^47^. For example, hydrophobic bile acids, not hydrophilic bile acids or conjugated bile acids are known to impair the mitochondrial electron transport chain resulting in oxidative stress. The potential toxicities of bile acids have been reported in the following order: deoxycholic acid > chenodesoxycholic acid > cholic acid > glycocholic acid > tauroursocholic acid^48^. In the present study, the most hazardous bile acids, including deoxycholic acid, chenodesoxycholic acid and cholic acid were increased, whereas, the relatively less toxic bile acids including glycocholic acid and some conjugated bile acids including tauroursocholic acid and sulfolithocholylglycine were decreased with CP treatment. In normal physical condition, the primary bile acids are synthesized from cholesterol by several rate-limiting enzymes including CYP7A1 and CYP8B1 in liver, repressed by FXR^37^,^49^. Most of bile acids can be reabsorbed into hepatocytes *via* both Na^+^ -dependent and Na^+^ -independent transports including NTCP and OATP^50^, and excreted into the bile canaliculus by transporters including BESP and MRP3. However, when CP was used, the expressions of FXR, CYP7A1 and NTCP were inhibited while the expression of MRP3 was activated^51^,^52^. The reason might be inferred that when CP was used, the organism showed adaptive modulation of bile acids to prevent bile acid overload by (a) suppressing bile acid *de novo* synthesis *via* FXR and CYP7A1 inhibition; (b) limiting bile acid re-absorption by NTCP inactivation; and (c) reducing bile acid accumulation in hepatocytes via basolateral excretion by MRP3 activation, which may also lead to damage to sinusoidal endothelial cells and hepatocyte plasma membrane.

When ginseng was co-administrated, the deoxycholic acid and chenodesoxycholic acid were decreased while the glycocholic acid was increased significantly with relieved oxidative stress respectively, which hinted that ginseng has ability to reduce the disordered homeostasis of bile acids. We further investigated the proteins associated to the synthesis, uptake, and exportation of bile acids. MRP3 was high expressed, the most hazardous bile acids were decreased and the hydrophilic bile acids were increased in liver, which indicated the decreasing of the bile salts in the hepatocytes. The expressions of the other proteins were also returned, which indicated the protective effects of ginseng on CP-induced liver damage.

GSH and phase II enzymes as well as transporters of bile acids^53^, including GCLC, GCLM, GS, GST, NTCP and MRP3^54^,^55^,^56^ were regulated by NRF 1 and NRF 2, the members of the cap ‘n’ collar-basic leucine zipper proteins. Thus, we further investigated the expressions of NRF 1 and NRF 2 when ginseng was co-administrated with CP. In the present study, ginseng treatment led to a significant increase in the protein expression of NRF 2 not NRF 1, indicating that NRF 2 might be a key factor in the protective effect of ginseng on CP-induced liver damage. Further study discovered that inhibiting NRF 2 reduced the expression of GCLC, GCLM, GS, GST, NTCP and MRP 3 and finally resulted in the depression of hepatopretective effect of ginseng in CP treated rats. All the observations verified that the ginseng-unregulated expression of GCLC, GS, GST, GST, NTCP and MRP3 by inducing the expression of NRF 2 in liver.

In summary, our results showed that ginseng exhibited a potent protective effect against CP-induced hepatotoxicity. The mechanisms involved can be attributed to modulating the disordered homeostasis of GSH and bile acid, and up-regulating GCLC, GS, GST, GST, NTCP and MRP3 by inducing the expression of NRF 2.

## Methods

### Chemicals and reagents

CP was purchased from Jiangsu Hengrui Medicine Co., Ltd. (Lianyungang, China). The ginseng sample (JSPACM-03-1) was collected from Jilin province and authenticated by Prof. Song-Lin Li *via* morphological and histological methods according to ginseng monograph in Chinese Pharmacopoeia (Version 2010). The voucher specimen was deposited in Department of Pharmaceutical Analysis and Metabolomics, Jiangsu Province Academy of Traditional Chinese Medicine. Reference compounds, including ginsenoside Re, Rg1, Rf, Ro, Rb1, Rc, Rb2, Rb3, Rd, 20(S)-Rg2, 20(S)-Rh2, 20(R)-Rg2 and 20(S)-Rg3, were purchased from Sichuan Victory Co. Ltd. (Chengdu, China). HPLC grade acetonitrile and methanol were purchased from Merck Company (Darmstadt, Germany). Antibodies for FXR, CYP7A1, CYP8B1, NTCP, OAT 2 and OAT 3, GST, BSEP, MRP 3, GCLM, GCLC, GS, NRF 1, NRF 2 and F4/80 were obtained from Santa Cruz Biotechnology (CA, USA). Brusatol was purchased from Beijing Zhili Biological Technology Co. Ltd. (Beijing, China). Ultra-pure water was prepared using Milli-Q SP system (Millipore, Bedford, MA, USA). All other reagents were of analytical grade and obtained from commercial sources.

### Drug preparation

CP was dissolved in normal saline solution for intraperitoneal administration (i.p.). Ginseng sample was accurately weighed, refluxed 75% ethanol at 85 °C for 3 h and then condensed under reduced pressure to produce residue. The contents of the main ginsenosides in the residue were measured quantitatively by our published UPLC-QTOF-MS method^57^, with the contents of Re, Rg1, Rf, Ro, Rb1, Rc, Rb2, Rb3, Rd, 20(S)-Rg2, 20(S)-Rh2, 20(R)-Rg2 and 20(S)-Rg3 to be 3018 μg/g, 7132 μg/g, 903 μg/g, 1823 μg/g, 2018 μg/g, 1021  μg/g, 859 μg/g, 142 μg/g, 1428 μg/g, 97 μg/g, 128 μg/g, 15 μg/g and 209 μg/g, respectively. The ginseng residue was subsequently suspended in 2% sodium carboxymethyl cellulose (CMC-Na) solution for intragastric administration (i.g.).

### Animal study and sample collection

Thirty-six male Sprague-Dawley rats (200 ± 20 g) were obtained from Shanghai Laboratory Animal Research Center (Shanghai, China) and maintained under controlled conditions (22–24 °C, 55–60% humidity and 12-hour light/dark cycle) with free access to standard food and water. All procedures were in accordance with the Regulations of Experimental Animal Administration issued by the Ministry of Science and Technology of the People’s Republic of China. All animal protocols were approved by the Animal Ethics Committee of Jiangsu Province Academy of Traditional Chinese Medicine.

After one week of acclimatization, the rats were randomly divided into six groups: blank group, CP treated group, CP and ginseng co-treated group, CP treated satellite group, CP and ginseng co-treated satellite group and inhibitor group. The blank group was i.g. 2 mL 1% CMC-Na solution and i.p. 0.5 mL normal saline, the CP treated group and CP treated satellite group were i.g. 2 mL 2% CMC-Na solution and i.p. CP solution (100 mg/kg), the CP and ginseng co-treated group and CP and ginseng co-treated satellite group were i.g. ginseng CMC-Na suspension (2 g/kg) and i.p. CP solution (100 m/kg), while the inhibitor group was i.g. ginseng CMC-Na suspension (2 g/kg) and i.p. CP solution (100 m/kg) 1 h after i.g. Brusatol (1 m/kg). All animals were treated once per day at 9:00 a.m. for ten consecutive days.

Blood samples 500 μL were collected into tubes via the ocular vein at 2 h after the last dosing. Serum samples were prepared by centrifuging the blood at 4000 rpm for 10 min at 4 ^o^C and stored at −80 ^o^C for further use. After the blood sampling, all animals were sacrificed by dislocated method. The liver tissues were removed and washed three times by normal saline for complete blood removal. Parts of the liver tissues were fixed in 10% formalin solution for histological study, and the remaining tissues were stored at −80 ^o^C for further use.

### Assessment of aminotransferase activities, LPO, GSH, antioxidant enzymes and inflammatory mediators

The activities of ALT, AST and ALP in serum and the levels or activities of MDA, GSH, SOD, CAT, GST, GPO and GR in liver were measured by commercially available kits (Nanjing Jiancheng Bioengineering Institute, Nanjing, China). The levels of IL6, IL8 and IL10 in serum were determined by rat enzyme-linked immuno-sorbent assay (ELISA) kits (Beijing Zhili Biological Technology Co. Ltd, Beijing, China), according to the manufacturer’s instructions.

### Histology and immunohistochemistry

Fixed liver tissues were subjected to dehydration in serial concentrations of alcohol and xylene followed by paraffin embedding. Four-micrometer serial sections were cut and stained with H&E, which was used to evaluate the structure of the hepatocytes. Histological examinations were performed using light microscopy (Zeiss Axio Observer A1). For immunohistochemical examination, deparaffinized sections were incubated with anti-rat F4/80 (1:1000 dilution) and appropriate biotinylated secondary antibodies, followed by the avidin-biotin-peroxidase complex. The immunoreactive signal was developed by color deposition, using diaminobenzidine as a substrate. Yellow material in the cytoplasm was considered to indicate positive cells. F4/80-positive macrophages were counted manually in five random fields under 400× magnification and expressed as the average number of positively labeled cells per unit area (mm^2^) of view.

### Metabolomic Study of Serum

#### Serum sample preparation

Aliquot of 150 μL serum was deproteinized by 600 μL methanol (HPLC grade, Merck, Darmstadt, Germany). The sample mixture was vortex-mixed for 3 min and centrifuged at 15000 rpm for 10  min at 4 ^o^C. The supernatant 650 μL was transferred into another tube and evaporated to dryness under a stream of nitrogen. The residue was reconstituted with 100 μL mobile phase, and an aliquot of 5 μL was injected into the UPLC-QTOF-MS/MS system for analysis.

#### UPLC-QTOF-MS/MS conditions

Chromatography was performed on an ACQUITY UPLC system (Waters Corp., Milford, MA, USA) with a conditioned autosampler at 8 ^o^C. The separation was carried out on a Waters Acquity HSS T3 (2.1 mm × 100 mm, 1.8 μm) and maintained at 35 ^o^C. The analysis was performed with gradient elution using (A) acetonitrile containing 0.1% formic acid and (B) water containing 0.1% formic acid as the mobile phase. The gradient program was: 0–1 min, 10% A; 1–3 min, linear increase 10–40% A; 3–8 min, linear increase 40–70% A; 8–10 min, linear increase 70–95% A; held at 95% A for 2 min and then immediately reduced to 10% A to equilibrate the column.

Mass spectrometry was performed on a Waters Synapt G2S Q-TOF-MS/MS (Micro mass MS Technologies, Manchester, UK) equipped with electrospray ionization source operating in both positive and negative modes. The nebulization gas was set at 1000 L/h at 450 °C, and the cone gas was set at 40 L/h. The capillary voltage and cone voltage were set at 3500 V and 45 V, respectively. Data were collected in centroid mode, and the MS^E^ approach was used. The energies for collision-induced dissociation (CID) were 6 V for the precursor ion and 10–40 V for fragmentation information. Leucine-enkephalin was used as the lock mass generating an [M + H]^+^ ion (*m/z* 556.2771) and [M − H]^−^ ion (*m/z* 554.2615) to ensure accuracy during the MS analysis.

#### Metabolomics analysis and data processing

The mass data acquired were imported to MakerLynx (version 4.1, Waters Corporation, Milford, USA) for peak detection and alignment. The retention time and *m/z* data for each peak were determined by the software. The main parameters were set as follows: retention time range 1–10 min, mass range 100–1000 Da, mass tolerance 0.1 Da, and noise elimination level 5. All data were normalized to the summed total ion intensity per chromatogram, and the resultant data matrices were introduced to EZinfo 2.0 software for PCA, PLS-DA and OPLS analysis after mean-centered and scaled to Pareto variance. The VIP value is a weighted sum of squares of the PLS weights, reflecting the relative contribution of each X variable to the model. The variables with VIP > 1 were considered to be influential for the separation of samples in the score plots generated from PLS-DA analysis.

### Western Blot

Liver tissue homogenates were prepared with RIPA Buffer and PMSF. Equal amount of denatured total protein (50 m) was separated by 8% SDS-PAGE and transferred onto PVDF membranes. The membranes were incubated with appropriate dilution of primary antibodies against FXR, CYP7A1, CYP8B1, NTCP, OAT 2, OAT 3, BSEP, MRP 3, GST, GCLM, GCLC, GS, NRF 1 and NRF 2, respectively. After incubated with proper rabbit anti-rat or goat anti-rat HRP-conjugated secondary antibodies, the intended proteins were detected using ECL kit (Invitrogen) and normalized to the corresponding β-actin expression.

## Additional Information

**How to cite this article**: Zhu, H. *et al*. Ginseng alleviates cyclophosphamide-induced hepatotoxicity *via* reversing disordered homeostasis of glutathione and bile acid. *Sci. Rep.* **5**, 17536; doi: 10.1038/srep17536 (2015).

## Figures and Tables

**Table 1:** Biochemical parameters of blank, CP treated and CP and ginseng co-treated groups (mean ± SD, n = 6).

**Groups**	**Liver**							**Serum**					
	**MDA (nmol/g protein)**	**GSH (nmol/mg protein)**	**SOD (U/mg protein)**	**CAT (U/mg protein)**	**GST (mU/mg protein)**	**GPO (U/mg protein)**	**GR (U/mg protein)**	**ALP (U/L)**	**AST (U/L)**	**ALT (U/L)**	**IL-6 (pg/mL)**	**IL-8 (pg/mL)**	**IL-10 (pg/mL)**
BK	4.32 ± 0.72	0.94 ± 0.14	105.2 ± 7.2	34.6 ± 5.4	4.16 ± 0.72	405.3 ± 21.9	120.9 ± 26.4	50.2 ± 8.2	30.7 ± 9.3	29.4 ± 4.32	32.4 ± 9.5	25.8 ± 5.0	61.5 ± 9.2
CP	8.54 ± 0.89**	0.54 ± 0.12*	84.8 ± 9.4*	24.8 ± 4.7*	2.03 ± 0.59**	298.1 ± 26.4**	137.6 ± 15.8*	123.3 ± 18.8**	84.2 ± 16.5*	61.4 ± 16.4*	123.6 ± 37.2**	82.3 ± 22.6*	21.4 ± 8.8**
CP+G	5.12 ± 0.75^#^	0.79 ± 0.09^#^	112.4 ± 8.2^#^	29.6 ± 3.7	3.83 ± 0.81^#^	386.4 ± 35.2^##^	131.7 ± 14.6	69.9 ± 10.3^#^	43.3 ± 12.7^#^	35.7 ± 26.8	54.1 ± 27.6^##^	66.4 ± 21.4	54.7 ± 10.7^#^

BK for blank group, CP for CP treated group and CP+G for CP and ginseng co-treated group. Compared with blank group, *p < 0.05 and **p < 0.01; Compared with CP group, ^#^p < 0.05 and ^##^p < 0.01.

**Table 2:** Endogenous metabolites of the serum from CP-induced rats and their identification results (mean ± SD, n = 6).

**No.**	**T**_**R**_**(min)**	**Molecular ion**	**Measured mass (Da)**	**VIP**	**Formula**	**Metabolites**	**Relative Intensity**			**HMDB**	**KEGG**
							**BK**	**CP**	**CP+G**		
M1	9.25	[M+H]^+^	524.3712	26.43	C_26_H_54_NO_7_P	LysoPC (18:0)	98.53 ± 6.81	145.51 ± 8.73**	107.49 ± 5.78^#^	10384	C04230
M2	7.50	[M+H]^+^	274.2743	7.39	C_11_H_23_N_5_O_3_	Valylarginine	18.47 ± 4.89	24.67 ± 5.79*	20.49 ± 4.31	29121	–
M3	7.59	[M+H]^+^	544.3393	10.49	C_28_H_50_NO_7_P	LysoPC (20:4)	247.83 ± 10.44	337.47 ± 14.73**	269.84 ± 12.83^##^	10395	C04230
M4	9.25	[M+H]^+^	546.3525	12.46	C_28_H_52_NO_7_P	LysoPC (20:3)	231.74 ± 9.83	296.58 ± 12.81*	287.37 ± 10.99	10394	C04230
M5	4.67	[M+H]^+^	400.3403	2.16	C_23_H_45_NO_4_	L-Palmitoylcarnitine	49.39 ± 4.28	87.33 ± 8.83**	52.17 ± 6.68^#^	00222	C02990
M6	5.11	[M+H]^+^	347.2217	3.22	C_21_H_30_O_4_	Cortexolone	24.36 ± 5.47	46.32 ± 8.99**	41.36 ± 6.84	00015	C05488
M7	5.64	[M+H]^+^	372.3108	4.17	C_21_H_41_NO_4_	Myristoylcarnitine	10.35 ± 3.28	17.93 ± 4.12*	15.49 ± 4.08	05066	–
M8	6.39	[M+H]^+^	426.3578	3.98	C_25_H_47_NO_4_	Oleoylcarnitine	37.72 ± 7.76	55.74 ± 8.58*	35.37 ± 5.76^##^	05065	–
M9	6.80	[M+H]^+^	468.3088	5.65	C_22_H_46_NO_7_P	Lyso PC (14:0)	184.38 ± 13.36	222.73 ± 17.41*	204.28 ± 12.84	10379	C04230
M10	7.75	[M+H]^+^	262.1160	4.59	C_11_H_19_NO_6_	Succinylcarnitine	9.76 ± 1.35	17.82 ± 2.32*	10.73 ± 2.21^#^	61717	–
M11	6.25	[M+H]^+^	318.3035	3.57	C_18_H_39_NO_3_	Phytosphingosine	14.86 ± 2.35	29.65 ± 3.44**	19.76 ± 3.32^#^	04610	C12144
M12	7.39	[M+H]^+^	520.3399	20.34	C_26_H_50_NO_7_P	LysoPC (18:2)	204.93 ± 18.48	154.32 ± 9.38**	229.39 ± 14.57^##^	10386	C04230
M13	7.82	[M+H]^+^	496.3398	9.465	C_24_H_50_NO_7_P	LysoPC (16:0)	358.39 ± 15.18	312.43 ± 12.19*	316.48 ± 14.74	10382	C00157
M14	9.74	[M+H]^+^	550.3869	14.58	C_28_H_56_NO_7_P	LysoPC (20:1)	198.42 ± 9.87	147.35 ± 8.47*	154.91 ± 9.15	10391	C04230
M15	8.42	[M+H]^+^	522.3553	11.49	C_26_H_52_NO_7_P	LysoPC (18:1(11Z))	152.88 ± 8.76	113.36 ± 7.90*	142.37 ± 10.27^#^	10385	C04230
M16	1.98	[M+H]^+^	249.0615	4.78	C_9_H_15_N_2_O_6_	γ-glutamylthreonine	34.43 ± 4.62	26.67 ± 5.32*	45.32 ± 5.44^##^	00713	–
M17	2.15	[M+H]^+^	251.0696	4.39	C_8_H_14_N_2_O_5_S	γ-glutamylcysteine	21.43 ± 3.25	15.38 ± 2.36*	19.48 ± 2.41^#^	01049	C00669
M18	4.20	[M-H]^−^	391.2847	8.94	C_24_H_40_O_4_	Chenodesoxycholic acid	4.78 ± 0.43	10.98 ± 1.03**	4.69 ± 0.76^#^	00951	C05465
M19	3.82	[M-H]^−^	391.2841	4.32	C_24_H_40_O_4_	Deoxycholic acid	18.47 ± 6.87	31.75 ± 7.86**	20.86 ± 8.86^#^	11637	C05122
M20	3.75	[M-H]^−^	514.2834	17.70	C_26_H_45_NO_7_S	Tauroursocholic acid	21.37 ± 4.90	12.47 ± 4.01**	19.35 ± 5.78^#^	00889	–
M21	1.90	[M-H]^−^	212.0029	12.67	C_8_H_7_NO_4_S	Indoxyl sulfate	13.25 ± 3.25	27.33 ± 5.24**	17.79 ± 4.38^#^	03309	–
M22	8.33	[M-H]^−^	283.2646	4.41	C_18_H_36_O_2_	Stearic acid	89.24 ± 6.96	149.75 ± 14.38**	107.84 ± 12.47^#^	00827	C01530
M23	3.12	[M-H]^−^	445.1892	6.33	C_24_H_30_O_8_	Estrone 3-glucuronide	14.23 ± 4.39	23.46 ± 5.49**	17.85 ± 5.77^#^	04483	C11133
M24	4.92	[M-H]^−^	329.2330	3.47	C_22_H_34_O_2_	Docosapentaenoic acid	12.93 ± 3.31	27.37 ± 3.38**	28.84 ± 6.49	06528	C16513
M25	3.88	[M-H]^−^	512.2680	10.25	C_26_H_43_NO_7_S	Glycolithocholate sulfate	9.73 ± 3.18	28.64 ± 6.42**	22.87 ± 6.43	02639	C11301
M26	6.07	[M-H]^−^	389.2687	4.68	C_24_H_38_O_4_	7a-Hydroxy-3-oxo-5β- cholanoic acid	3.58 ± 0.67	5.48 ± 0.89*	4.46 ± 0.87	00503	C00281
M27	5.39	[M-H]^−^	407.2797	4.60	C_24_H_40_O_5_	Cholic acid	5.63 ±1.01	8.64 ± 0.82*	7.52 ± 1.56	02639	C11301
M28	6.71	[M-H]^−^	429.3010	3.52	C_27_H_42_O_4_	7a-Hydroxy-3-oxo-4- cholestenoate	6.87 ± 0.95	9.32 ± 0.99*	7.10 ± 1.02^#^	12458	C00157
M29	3.88	[M-H]^−^	512.2676	5.42	C_26_H_43_NO_7_S	Sulfolithocholylglycine	46.25 ± 6.42	24.17 ± 4.32**	32.29 ± 4.48^#^	00619	C00695
M30	2.02	[M-H]^−^	203.0836	2.98	C_11_H_12_N_2_O_2_	Tryptophan	16.48 ± 3.11	9.78 ± 1.07*	10.74 ± 1.21^#^	00929	C00525
M31	4.82	[M-H]^−^	464.3012	23.83	C_26_H_43_NO_6_	Glycocholic acid	31.03 ± 5.11	24.56 ± 6.47*	29.47 ± 8.79	00138	C01921
M32	8.62	[M-H]^−^	566.3456	23.93	C_30_H_50_NO_7_P	LysoPC (22:6)	197.33 ± 12.54	135.83 ± 11.25*	147.78 ± 10.83	10404	C04230
M33	4.43	[M-H]^−^	188.0724	4.44	C_11_H_11_NO_2_	3-Indolepropionic acid	12.44 ± 1.24	8.49 ± 0.87*	10.82 ± 0.90^#^	02302	–

BK for blank group, CP for CP treated group and CP+G for CP and ginseng co-treated group. Compared with BK, *p < 0.05 and **p < 0.01; Compared with CP, ^#^p < 0.05 and ^##^p < 0.01.
